# Single breath-hold 3D measurement of left atrial volume using compressed sensing cardiovascular magnetic resonance and a non-model-based reconstruction approach

**DOI:** 10.1186/s12968-015-0147-8

**Published:** 2015-06-11

**Authors:** Orestis Vardoulis, Pierre Monney, Amit Bermano, Amir Vaxman, Craig Gotsman, Janine Schwitter, Matthias Stuber, Nikolaos Stergiopulos, Juerg Schwitter

**Affiliations:** Laboratory of Hemodynamics and Cardiovascular Technology, Institute of Bioengineering, Swiss Federal Institute of Technology, Ecole Polytechnique Fédérale de Lausanne, Lausanne, Switzerland; Division of Cardiology and Cardiac MR Center, University Hospital of Lausanne (CHUV), Lausanne, Switzerland; Department of Radiology, University Hospital and University of Lausanne, Lausanne, Switzerland; Center for Biomedical Imaging, Lausanne, Switzerland; Computer Graphics lab, ETH Zurich & Disney Research Zurich, Zurich, Switzerland; Geometric Modeling and Industrial Geometry group, Vienna University of Technology, Vienna, Austria; Jacobs Technion-Cornell Institute at Cornell Tech, New York, USA; University of Fribourg, Biomedical Sciences, Fribourg, Switzerland

## Abstract

**Background:**

Left atrial (LA) dilatation is associated with a large variety of cardiac diseases. Current cardiovascular magnetic resonance (CMR) strategies to measure LA volumes are based on multi-breath-hold multi-slice acquisitions, which are time-consuming and susceptible to misregistration.

**Aim:**

To develop a time-efficient single breath-hold 3D CMR acquisition and reconstruction method to precisely measure LA volumes and function.

**Methods:**

A highly accelerated compressed-sensing multi-slice cine sequence (CS-cineCMR) was combined with a non-model-based 3D reconstruction method to measure LA volumes with high temporal and spatial resolution during a single breath-hold. This approach was validated in LA phantoms of different shapes and applied in 3 patients. In addition, the influence of slice orientations on accuracy was evaluated in the LA phantoms for the new approach in comparison with a conventional model-based biplane area-length reconstruction. As a reference in patients, a self-navigated high-resolution whole-heart 3D dataset (3D-HR-CMR) was acquired during mid-diastole to yield accurate LA volumes.

**Results:**

Phantom studies. LA volumes were accurately measured by CS-cineCMR with a mean difference of −4.73 ± 1.75 ml (−8.67 ± 3.54 %, r^2^ = 0.94). For the new method the calculated volumes were not significantly different when different orientations of the CS-cineCMR slices were applied to cover the LA phantoms. Long-axis “aligned” vs “not aligned” with the phantom long-axis yielded similar differences vs the reference volume (−4.87 ± 1.73 ml vs −4.45 ± 1.97 ml, *p* = 0.67) and short-axis “perpendicular” vs “not-perpendicular” with the LA long-axis (−4.72 ± 1.66 ml vs −4.75 ± 2.13 ml; *p* = 0.98). The conventional bi-plane area-length method was susceptible for slice orientations (*p* = 0.0085 for the interaction of “slice orientation” and “reconstruction technique”, 2-way ANOVA for repeated measures). To use the 3D-HR-CMR as the reference for LA volumes in patients, it was validated in the LA phantoms (mean difference: −1.37 ± 1.35 ml, −2.38 ± 2.44 %, r^2^ = 0.97). Patient study: The CS-cineCMR LA volumes of the mid-diastolic frame matched closely with the reference LA volume (measured by 3D-HR-CMR) with a difference of −2.66 ± 6.5 ml (3.0 % underestimation; true LA volumes: 63 ml, 62 ml, and 395 ml). Finally, a high intra- and inter-observer agreement for maximal and minimal LA volume measurement is also shown.

**Conclusions:**

The proposed method combines a highly accelerated single-breathhold compressed-sensing multi-slice CMR technique with a non-model-based 3D reconstruction to accurately and reproducibly measure LA volumes and function.

## Background

Left atrial (LA) dilatation is associated with a large variety of cardiac diseases and is the result of chronic volume and/or pressure overload of the LA. It has been associated with the severity of left ventricular (LV) diastolic dysfunction in population studies [1] and it is a recognized adverse prognostic marker in several disease states, such as heart failure [2, 3], hypertension [4], myocardial infarction [5, 6], hypertrophic cardiomyopathy [7], or mitral valve disease [8-10].

First data regarding the prognostic value of LA size were obtained from population studies only measuring the antero-posterior diameter of the LA with M-mode echocardiography [11]. However, LA enlargement does not occur in a uniform fashion in all three directions in space with disease, and single plane measurements may be insensitive to detect early LA dilatation. LA diameters or areas have been proposed for everyday use, but their correlations with volumes were variable [12, 13]. Also, biplane Simpson’s or area-length methods for LA volume calculations rely on geometric assumptions and there is evidence that they underestimate the true LA volume [14, 15]. Finally, several studies indicate that 3D-acquired volumes are associated with a higher accuracy and, most importantly, are better associated with outcome [10, 16-19]. Accordingly, the assessment of LA volume is now recommended in clinical routine [20].

Cardiovascular magnetic resonance (CMR) is considered as the reference method for volume measurements of cardiac chambers using the multiple slice technique [21, 22]. However, multi-breathhold multi-slice acquisitions are somewhat time-consuming and in addition, they may suffer from registration errors, if the patient does not hold his breath at identical diaphragmatic positions. To reduce the number of breath-holds, undersampling is an option which can be compensated for by analysis approaches that are model-based. A time-efficient CMR method to precisely measure the LA volume without the need for repetitive breath-holds and without requiring geometric assumptions for reconstruction is highly desirable. In the past years, accelerated CMR techniques emerged exploiting temporo-spatial correlations [23] or spatially localized excitations [24] to allow for substantial undersampling and acceleration. Recently, a very fast, i.e. a so-called compressed sensing MR acquisition technique [25] was applied to the LV which was combined with a model-based analysis tool to extract volumes from the multi-slice data sets [26]. Here, we propose to measure LA volumes with such a highly accelerated compressed sensing CMR technique in order to acquire up to five non-parallel slices covering the LA with high temporal and spatial resolution in a single breath-hold and to combine it with a novel “non model-based” reconstruction strategy to extract LA volumes. From these data, time-volume curves of the LA can be derived which might be advantageous as dynamic LA volume changes provide an incremental prognostic value over simple LA volume measurements [27].

Accordingly, the aim of the current study was to combine a compressed-sensing accelerated CMR technique for LA volume measurements with a novel 3D non-model-based reconstruction algorithm and to evaluate the accuracy of this approach for the quantification of LA volumes in phantoms and patients.

## Methods

The accuracy of the novel compressed-sensing based technique (CS-cineCMR) was validated with LA-shaped non-moving phantoms of known volumes. Furthermore, it was evaluated in these phantoms, whether the slice orientations covering the LA phantoms impact on the accuracy of the LA volume measurements.

Subsequently, the CS-cineCMR technique was assessed in patients. For the patients, a 3D high-resolution CMR acquisition (3D-HR-CMR) was performed, that measures the LA volume with high spatial resolution at one time point in the cardiac cycle thereby yielding the in-vivo reference LA volume. To this end, the accuracy of this 3D-HR-CMR acquisition was validated in the LA phantoms. In the patients, the LA volumes determined with the 3D-HR-CMR technique (=reference volume in patients) were compared with the LA volumes determined by the CS-cineCMR technique. Finally, an intra- and inter-observer analysis was performed on 10 datasets to assess the impact of semi-automatic segmentation on the volume estimation and the robustness of the method.

### A. Phantom experiments

**Fig. 1:**
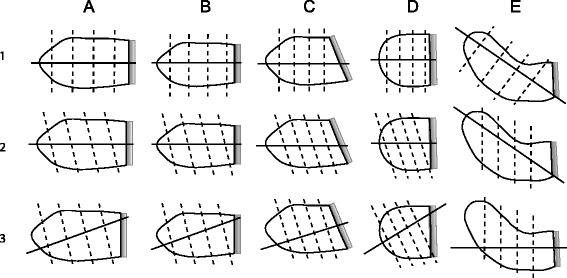
Presents the LA phantom shapes and the corresponding acquisition strategies that were utilized for the assessment of the CS-cineCMR sequence and reconstruction methods. The mitral valve is represented by the fading gray shape on the right. The large and small volumes of shapes **a** and **b** correspond to systolic and diastolic phases, respectively. Shape **c** represents a LA with oblique connection to the LV, which is occasionally found in patients with a hypertrophied LV and/or obesity. Phantom shape **e** (=curved) mimics impression of an enlarged LA by the aortic root. To complete the spectrum of LA shapes a spherical phantom (**d**) was also analyzed. **a**) Oval Big (= LA at end-systole), **b**) Oval small (=LA at end-diastole), **c**) Oval oblique, **d**) Spherical and **e**) Curved (=LA compression by aortic root). Row 1 corresponds to long-axis acquisitions aligned with the long-axis of the atrium (and with short-axis planes perpendicular to acquisition long-axis). Row 2 corresponds to long-axis acquisitions aligned with the long-axis of the atrium, but with short-axis planes non-perpendicular to the atrium long-axis. Row 3 corresponds to long-axis acquisitions not-aligned with the long-axis of the atrium and with short-axis planes perpendicular to the acquired long-axis

Five LA phantoms were created to resemble a set of typical LA morphologies and volumes (oval small (diastolic), oval large (systolic), oblique, spherical, curved) as shown in Fig. 1. The phantoms were made of manually carved Solanum Tuberosum L. These phantom volumes were measured with the water displacement method to yield the reference volumes required to validate the CS-cineCMR and the 3D-HR-CMR techniques. With these phantoms it was also evaluated whether various slice orientations of the CS-cineCMR technique influence the accuracy of LA volume measurements when using the novel 3D non-model-based reconstruction or a conventional model-based bi-plane area-length reconstruction.

### MR imaging

#### - Compressed-sensing cine CMR technique (CS-cineCMR)

Time-resolved images of the LA phantoms were acquired with the novel prototype compressed sensing CMR sequence on a 1.5 T MR clinical scanner (Magnetom Aera, 1.5 T, Siemens, Erlangen, Germany). This method is based on exploiting the sparsity promoting principle along the phase encoding and the time directions. The sparse data representation was achieved through the redundant Haar wavelet transform. The required incoherent sampling was implemented by an acquisition pattern that incorporates variable density sub-sampling with an increasing rate towards the k-space periphery. Furthermore, an effect similar to that of partial Fourier was obtained using a data-skip scheme, asymmetrical to k-space center. For the case of sparse cine-CMR imaging, a pseudo-random offset is applied in a frame-to-frame basis resulting in temporal incoherence. Pairing was also applied, to avoid eddy current effects in the SSFP acquisitions [28]. As previously described in [29], image reconstruction was performed with an iterative SENSE approach [30] and a L1-regularization, while coil sensitivity maps were calculated from the temporal average of all input data. A modified version of the FISTA algorithm was implemented and combined with the Haar wavelet transform [31]. In addition, the iterative reconstruction was performed after Fourier transformation along the readout direction, which allows a high degree of parallel processing. The whole image reconstruction process was performed on conventional computational hardware that included an eight-core processor unit (Intel® Xeon® E5540, 2.53 GHz, Intel Corporation, Santa Clara, CA, USA).

The multi-slice prospectively ECG-triggered compressed sensing acquisition was performed during a simulated heart rate of 60 bpm and acquired two long-axis and three short axis slices of the LA phantoms in 20 heart beats. Thirty elements of anterior and posterior phased-array coils were activated for signal reception. Imaging parameters were as follows: acceleration factor: 11.0, temporal/spatial resolution: 30 ms/1.5x1.5 mm^2^, slice thickness: 6 mm, flip angle: 70°, TE/TR: 1.23/2.89 ms and Bandwidth: 875 Hz/Px. The cine loop for each slice prospectively covered slightly more than one R-R interval to ensure covering a full cardiac cycle with this prospective acquisition scheme resulting in ten profiles per heart phase. The multislice CS-cineCMR technique offers the possibility to align the acquired slices at any plane orientation resulting in a variety of imaging strategies. Therefore, we further explored the possible impact of various slice orientations on the volumetric results. Figure 1 presents the 5 LA phantom shapes that were used and the corresponding planning strategies that were tested.

#### - 3D High-Resolution CMR (3D-HR-CMR)

Data acquisition was performed with a prototype self-navigated isotropic 3D balanced steady state free-precession (bSSFP) sequence with a radial readout following a spiral phyllotaxis pattern [32] that was adapted for self–navigation as previously described [33, 34]. The 3D-HR-CMR sequence is segmented and ECG-triggered and was run with a simulated heart rate of 60 bpm with the following acquisition parameters: TR/TE 3.1/1.56 ms, FOV 190 mm^3^, matrix 208^3^, acquired isotropic voxel size 0.91 mm, radiofrequency (RF) excitation angle 115°, receiver bandwidth 890 Hz/Px, and a trigger delay of 600 ms. Throughout the acquisition, a total number of 11’687 radial readouts were recorded.

Volumes of the LA phantoms were measured on the 3D-HR-CMR datasets using the GT-Volume software (GyroTools Ltd, Switzerland). The volume measurement was performed using the multiple-slice technique by manually tracing the LA contours on the high-resolution axial slices.

### B. Study subjects

Three subjects referred for a clinically indicated CMR examination were included in the study. Table 1 presents their clinical characteristics. The subjects were studied on the same 1.5 T MR scanner as used for the phantom studies. Both, the 3D-HR-CMR and the CS-cineCMR technique were applied at the end of the routine CMR protocol. The study was approved by the local ethics committee and patients gave written informed consent before study participation.

**Table 1:** Patient demographics

	Patient 1^a^	Patient 2^b^	Patient 3^c^	
Age (y)	23	53	80	
Gender	male	male	male	
Weight (kg)	72	77	74	
Height (cm)	177	170	175	
BMI (kg/m^2^)	23.0	24.2	24.2	
BSA (m^2^)	1.88	1.90	1.89	

^a^Operated for atrial septal defect at age six. Follow-up imaging because of persistent right ventricular enlargement

^b^Ischemic heart disease (three-vessels coronary heart disease)

^c^Severe valvular heart disease: aortic stenosis considered for aortic valve replacement. Previous mitral valve replacement with a mechanical prosthesis at age 71 and now prosthesis dysfunction with moderate to severe paravalvular leak

### CMR data acquisition

#### - CS-cineCMR

One LA long-axis slice was planned on a standard long-axis 3-chamber view of the LV in an orientation crossing the center of the mitral plane and the roof of the LA. The other LA long-axis slice was planned perpendicular to the previous long-axis slice. The three short-axis LA slices were oriented perpendicular to the 2 long-axis slices and were placed at quasi-equal distances in the proximal, mid, and distal portion of the LA.

To assess the image quality obtained with the CS-cineCMR imaging, the LA endocardial border sharpness (EBS) was quantified at end diastole (minimal LA volume) and end-systole (maximal LA volume) in both long axis and short axis view in three patients (shown in Fig. 6) as described in [35]. For each cine slice, the EBS was calculated as the average of 8 EBS profiles measured along the circumference of the LA wall, a higher EBS value (expressed as 1/Pixels) indicating a higher level of border sharpness. The mean EBS of the compressed sensing cine images was compared to the EBS of corresponding standard b-SSFP cine images.

#### - 3D-HR-CMR

For this acquisition, the trigger delay was set to the most quiescent mid-diastolic period by the operator through visual inspection of a cine acquisition in 4-chamber long-axis orientation. Volumes of the LA were measured on the 3D-HR-CMR datasets using the GT-Volume software (GyroTools Ltd, Switzerland) as described above. The LA appendage as well as the ostia of the pulmonary veins were excluded from the LA volume.

#### 3D non-model-based reconstruction

##### - Segmentation of the CS-cineCMR images

The patient and phantom LA contours were delineated on all the acquired 2D-slices with an expanding balloon method that utilizes a gradient-based edge detection algorithm as implemented in the open access ITK-SNAP software [36]. The segmentation process is initialized by a set of manually placed segmentation balloons within the region of interest (ROI) and then the software expands the initial boundaries based on the image intensity values. The 2D time-resolved CS-cineCMR data were analyzed with the 3D snake tool where “time” was considered as the z-axis. This strategy was chosen to achieve better continuity on the delineation results in-between consecutive timeframes, compared to processing each time step separately. An experienced ITK-SNAP user performed the segmentations and, as for 3D-HR-CMR datasets, the LA appendage and the ostia of the pulmonary veins were excluded from the segmented LA surface. The automated segmentation results were visually inspected and any artifacts or incomplete exclusion of the left atrial appendage/pulmonary veins were corrected with the manual segmentation tool provided by the software. Finally, the segmentation information (3D binary masks) were saved in the NIFTI file-format (*.nii) for further processing of the segmented ROI’s.

##### - Point cloud generation

The point clouds describing the 3D surfaces of the atria were generated based on the segmented contours with a dedicated Matlab script (MathWorks, Natick, MA). Initially the NIFTI segmentation files were loaded and the corresponding 3D segmentation data were broken down into 2D time-resolved binary masks. For each slice location the DICOM file metadata were processed in order to transfer the LA contours into global coordinates. This was performed by creating a transformation matrix, using the provided direction cosines and the location of the left uppermost image pixel in global coordinates. For each slice and timeframe, the contour data were transformed to the global coordinate system and smoothed with a robust smoothing algorithm for gridded data [37].

##### - 3D surface reconstruction from CS-cineCMR acquisition

The segmented and smoothed contours were reconstructed using a method based on Bermano and coworkers for the extraction of triangle meshes from planar contours as cross-sectional slices [38]. The algorithm proceeds in the following steps: first, we arrange the planes on which the contours reside as a collection of three-dimensional cells. Second, we define a binary indicator function such that the inner region on the plane that each contour encompasses is considered as “inside” (then having the value one). The outside is defined accordingly with the value − one. The contour itself is then implicitly the zero set of this indicator. Third, we interpolate the indicator function from the planes into every point in three dimensions using Mean-Value coordinates [39]. Finally, we extract a triangle mesh from the zero set of the interpolated function using the mesh generation package provided by CGAL (Computational Geometry Algorithms Library, http://www.cgal.org). This is done for every given set of contours, in every time frame and the volume of that time point is computed by the volume of the resulting triangle mesh without being based on a geometrical model assumption. The algorithm is implemented in C++, and the interpolation algorithm is aided by parallel GPU computation with CUDA.

### Model-based LA volume calculation

The performance of the 3D non-model-based reconstruction was compared with a model-based approach, i.e. the bi-plane area-length method which is recommended for quantification of LA volumes by echocardiography [20]. The bi-plane area-length method was applied to the CS-cineCMR data acquired in the phantoms. In order to account for different LA shapes, the modified equation was used where the minimum LA length “L” in the equation (measured either in the area_4ch_ and area_2ch_ plane) is replaced by the mean of the lengths measured on the two orthogonal long-axis planes yielding the formula: LA volume = (0.848 × area_4ch_ × area_2ch_)/([length_2ch_ + length_4ch_]/2) [15]. Length_4ch_ and length_2ch_ were measured as the maximum distance from the mitral valve mid-position (grey line on the phantoms as illustrated in Fig. 1) to the opposite wall.

### C. Intra and inter-observer variability analysis

Intra- and inter-observer reproducibility analysis of LA volume measurement was performed on ten patients to assess the robustness of the semi-automatic segmentation method. For each compressed sensing cine dataset, the segmentation was performed twice by the same observer P.M. (Observer 1A and Observer 1B results) to assess intra-observer variability and once by the observer O.V. (Observer 2 results). Observer 1 and Observer 2 results were compared to assess inter-observer variability.

### Statistics

Bland-Altman analysis were used to compare the reference LA volumes of the phantoms with the volumes determined by the CS-cineCMR and the 3D-HR-CMR techniques. For these comparisons linear regression analyses are also provided. Analysis of the influence of imaging strategy on CS-cineCMR accuracy was performed with the paired *t*-test (Matlab, Mathworks, Natick,USA). The volume differences of the strategies in Fig. 1 Row 1 and 2 were compared against Row 3 to assess the effect of different long-axis alignments with the LA long-axis. The volume differences of Fig. 1, Row 2 were compared against Row 1 and Row 3 to assess the effect of perpendicularity of short-axis planes with respect to the LA long-axis. The effect of long-axis alignment was also evaluated for the model-based, i.e. the bi-plane area-length approach, in comparison with the non-model based 3D reconstruction using a two-way ANOVA for repeated measures with within factors being “imaging strategy” (aligned vs not-aligned) and “reconstruction technique” (3D-non-model-based vs model-based biplane area-length method). Intra- and inter-observer agreement for LA volume measurement in patients was also assessed with Bland-Altman analysis of mean difference and standard deviation of differences.

## Results

### A. LA phantom studies

#### CS-cineCMR versus reference LA volumes

**Fig. 2:**
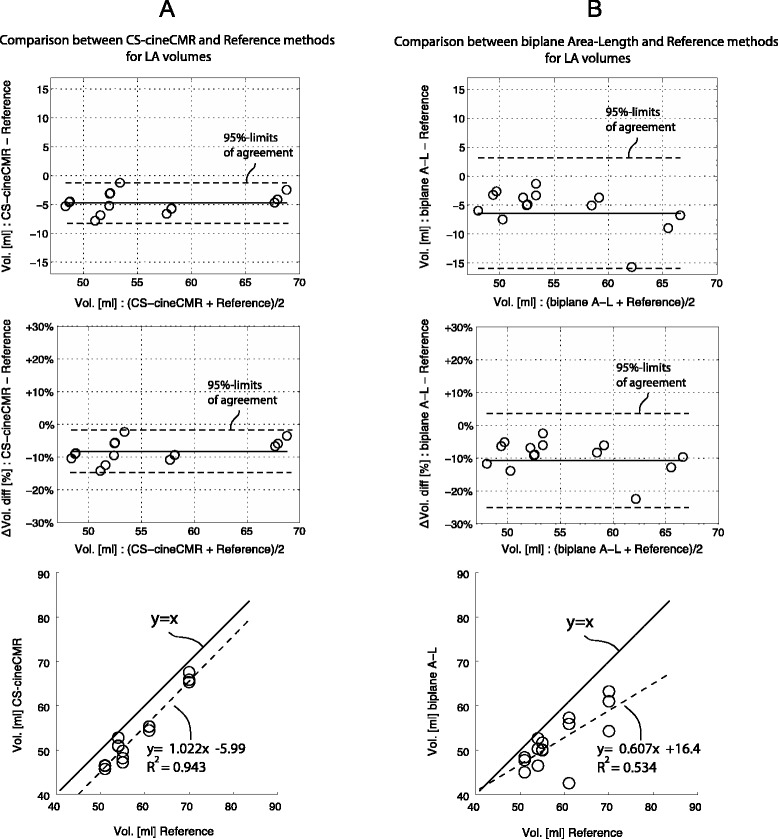
**a**. Comparison: CS-cineCMR versus reference (water displacement). Bland-Altman and regression analysis for the comparison between reference volumes and CS-cineCMR. In the Bland- Altman plots, the middle (solid) line represents the mean of differences and the two dashed lines represent ±2*SD. **b**. Comparison: biplane Area-Length versus reference (water displacement). Bland-Altman and regression analysis for the comparison between reference volumes and 3D-HR-CMR. In the Bland- Altman plots, the middle (solid) line represents the mean of differences and the dashed lines represent ±2*SD

LA phantom volumes were measured by CS-cineCMR and compared against the corresponding reference volumes measured with the water displacement method. The Bland-Altman analysis (Fig. 2a) resulted in an overall mean difference of −4.73 ± 1.75 ml (−8.8 ± 3.3 %; p < 0.01). The correlation between the two techniques was high with r^2^ equal to 0.94 and a slope close to one (Fig. 2a). Detailed results are given in Table 2.

**Table 2:** Comparison between the reference LA volumes the CS-cineCMR combined with the 3D non-model-based and the model-based bi-plane area-length reconstruction. Acquisition strategies A1 to E3 are explained in Fig. 1

	Acquisition	CS-cineCMR 3D non-model based Recon.	Reference	Diff	Diff [%]
		Volume [ml]	Volume [ml]	Volume [ml]	
Oval big	A1	51.0	54.0	−3.0	−5.8 %
	A2	50.9		−3.1	−6.0 %
	A3	52.8		−1.2	−2.3 %
Oval small	B1	45.7	51.0	−5.3	−11.0 %
	B2	46.4		−4.6	−9.5 %
	B3	46.5		−4.5	−9.2 %
Oval oblique	C1	55.3	61.0	−5.7	−9.8 %
	C2	55.3		−5.7	−9.9 %
	C3	54.4		−6.6	−11.5 %
Spherical	D1	48.2	55.0	−6.8	−13.3 %
	D2	47.2		−7.8	−15.3 %
	D3	49.8		−5.2	−10.0 %
Curved	E1	65.8	70.0	−4.2	−6.1 %
	E2	67.5		−2.5	−3.6 %
	E3	65.3		−4.7	−6.9 %
All	-	53.5 ± 7.3	58.2 ± 7.0	−4.7 ± 1.8	−8.7 ± 3.5 %
Oval big	A1	52.67	54.0	−1.33	−2.5 %
	A2	50.29		−3.71	−7.1 %
	A3	46.51		−7.49	−14.9 %
Oval small	B1	48.38	51.0	−2.62	−5.3 %
	B2	47.76		−3.24	−6.6 %
	B3	45.03		−5.97	−12.4 %
Oval oblique	C1	55.92	61.0	−5.08	−8.7 %
	C2	57.28		−3.72	−6.3 %
	C3	42.57		−18.43	−35.6 %
Spherical	D1	50.10	55.0	−4.90	−9.3 %
	D2	51.66		−3.34	−6.3 %
	D3	49.96		−5.04	−9.6 %
Curved	E1	61.02	70.0	−8.98	−13.7 %
	E2	63.23		−6.77	−10.2 %
	E3	54.29		−15.71	−25.3 %
All	-	51.8 ± 5.8	58.2 ± 7.0	−6.4 ± 4.8	−11.5 ± 8.5 %

For the model-based, i.e. the bi-plane area-length method applied to the CS-cineMR data, true LA phantom volumes were underestimated by −6.3 ± 4.8 ml (−11.4 ± 8.6 %, p < 0.0001) and linear correlation was low with r^2^ of 0.54 and a slope of 0.61 (see also Fig. 2b).

### Impact of the slice orientation on volume measurement

It was also investigated whether the alignment of the acquired long-axis with respect to the LA long-axis could have an influence on the volumetric results. When the LA volumes of the two groups with the long-axis “aligned” and “not-aligned” with the phantom LA long-axis were compared with the reference LA volumes no difference was found with −4.87 ± 1.73 ml and −4.45 ± 1.97 ml, respectively (p = 0.67). Similarly, when the LA volumes of the two groups with “perpendicular” and “non-perpendicular” short-axis acquisitions were compared with the true references LA volumes, no difference was found with −4.72 ± 1.66 ml and −4.75 ± 2.13 ml, respectively (p = 0.98).

**Fig. 3:**
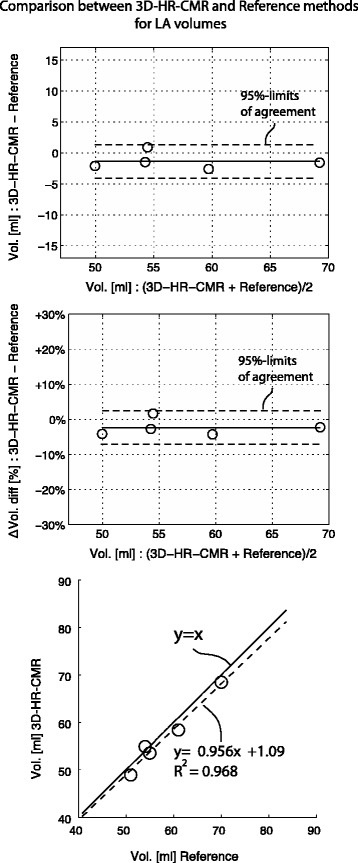
Comparison: 3D-HR-CMR versus reference (water displacement)

Conversely, for the bi-plane area-length method, long-axis alignment was relevant for the LA volume calculations. The difference to the reference LA volumes was −4.36 ± 2.19 ml (−7.60 ± 3.10 %) when the long-axis acquisition was “aligned” with the long-axis of the LA, but increased to −10.52 ± 6.11 ml (−19.60 ± 10.70 %), when “not-aligned”. The two-way ANOVA for repeated measures demonstrated a significant interaction between the factors “alignment/not-alignment” and “non-model-based/model-based reconstruction” for the calculation of LA volumes vs the reference phantom volume (difference in ml: *p* = 0.0085; difference in %: *p* = 0.0053, Fig. 3).

### LA phantoms 3D high-resolution versus reference volume

**Fig. 4:**
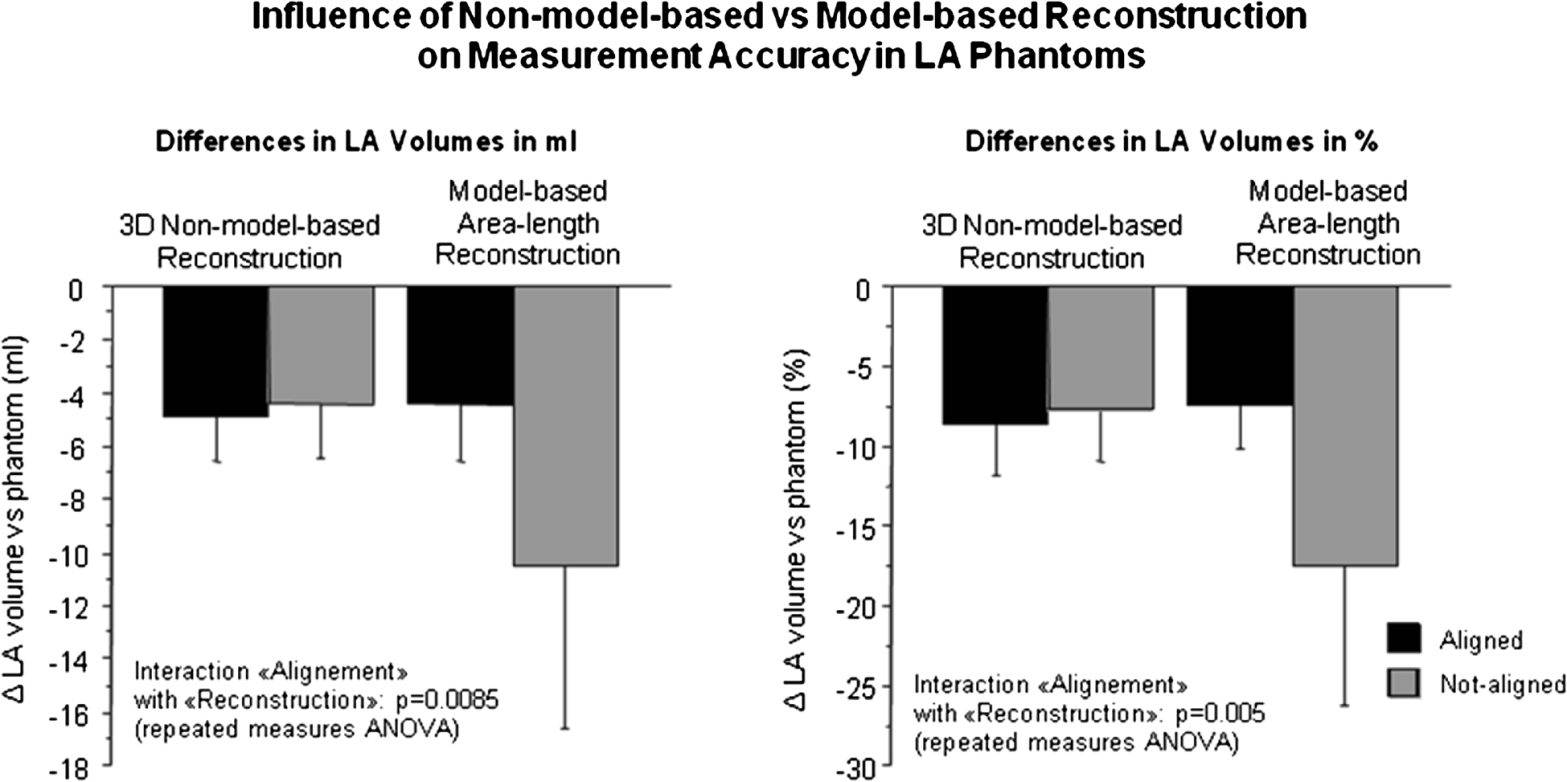
For the CS-cineCMR technique combined with the 3D non-model-based reconstruction, no differences are observed between acquisitions aligned with the long-axis of the LA or not, nor for acquisitions with short-axis perpendicular to the long-axis of the LA or not. However, for the model-based bi-plane area-length method, significant differences exist between the acquisitions with alignment of the long-axis or not (*p* = 0.0085 for differences in ml; *p* = 0.0053 for differences in %, for interaction in the two-way repeated measures ANOVA)

There was good agreement between the volumes measured by 3D-HR-CMR and the reference volumes measured by the displacement method. The mean difference was −1.37 ± 1.35 ml (−2.38 ± 2.44 %, *p* = 0.08, Fig. 4). The correlation between both techniques for volume measurements was also good with r^2^ equal to 0.97 and a slope close to 1 (Fig. 4). Table 3 summarizes the data.

**Table 3:** Comparison of the 3D-HR-CMR volumes against the reference LA phantom volumes (water displacement method)

	3D-HR-CMR	Reference	Diff	Diff [%]
	Volume [ml]	Volume [ml]	Volume [ml]	
Oval big	54.9	54.0	0.9	1.7 %
Oval small	48.9	51.0	−2.1	−4.2 %
Oval oblique	58.4	61.0	−2.6	−4.4 %
Spherical	53.5	55.0	−1.5	−2.8 %
Curved	68.4	70.0	−1.6	−2.3 %

### B. Patient studies: In vivo evaluation of CS-cineCMR

**Fig. 5:**
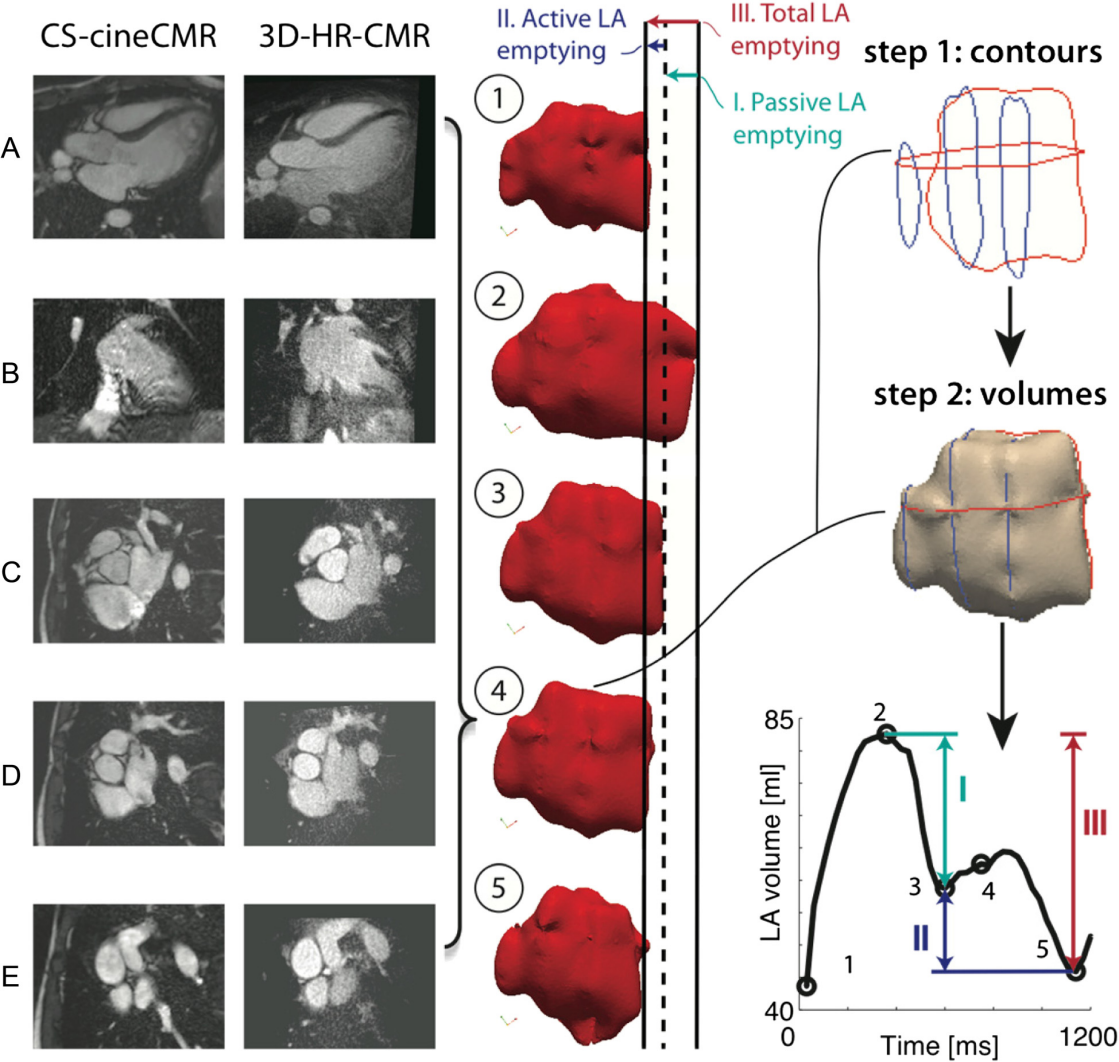
Left: Comparison between CS-cineCMR and 3D-HR-CMR images for the most quiescent mid diastolic phase (time-point four). Images **a** and **b** correspond to the long axis and images **c**, **d** and **e** to the short axis. Middle: Five snapshots of the atrial function with emphasis on the mitral plane motion. Dark line corresponds to the location of the mitral valve plane at time-point 1. Right: Visual representation of the method as applied for the volume reconstruction of the atrium in time-point four and graph showing the time-volume curve for patient one, where (I) corresponds to passive LV filling, (II) corresponds to active LV filling and (III) corresponds to overall LA emptying

**Fig. 6:**
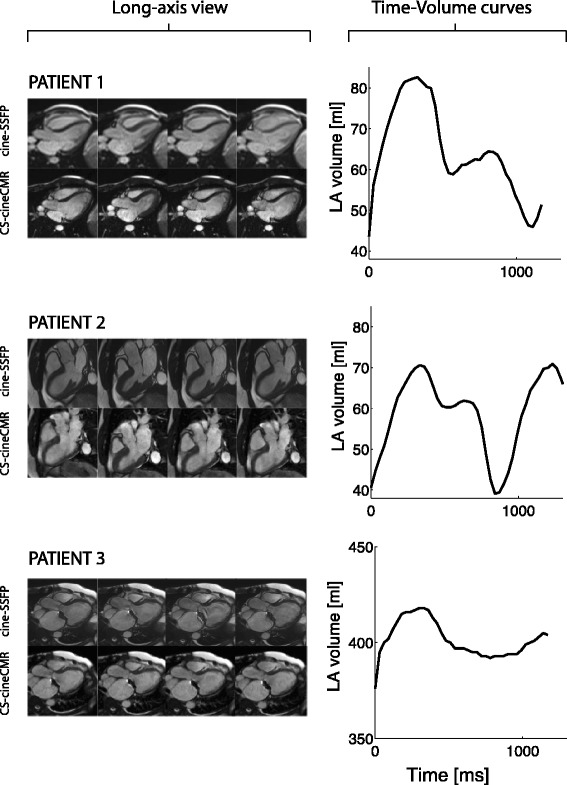
Left column: Long-axis view for the three patients. Right column: Corresponding time – LA volume curves generated from the CS-cineCMR acquisitions

Given the good agreement between the 3D-HR-CMR and the water displacement technique for the LA phantom volumes, the 3D-HR-CMR method was used as the reference for evaluating the in-vivo accuracy of the novel CS-cineCMR technique in patients. Results are presented in Table 4. LA volumes calculated from the 3D-HR-CMR data and the CS-cineCMR data showed good agreement. The average difference for the three in vivo cases was −2.66 ± 6.5 ml (3.0 %, *p* = 0.55). As shown on Fig. 5 the non-parallel reconstruction from CS-cineCMR data not only provides a static volume measurement of the LA, but also the dynamic LA volume changes over time during the whole heart cycle allowing for calculation of indexes of LA function. Examples of LA volume-time curves are shown in Fig. 6 and the corresponding functional indices of the LA (measured with the new approach) and the LV (measured by conventional acquisitions and Simpson’s rule) are given in Table 5.

**Table 4:** Comparison of the LA volumes of the CS-cineCMR and 3D-HR-CMR acquisitions in the three patients

	CS-cineCMR	3D-HR-CMR(ref)	Diff	Diff [%]
	Volume [ml]	Volume [ml]	Volume [ml]	
Patient 1	62.5	59.0	3.5	5.7 %
Patient 2	61.5	71.0	−9.5	−14.3 %
Patient 3	395.0	397.0	−2.0	−0.5 %

**Table 5:** LV and LA hemodynamics and morphology

Left ventricle				
	Patient 1 (23 years)	Patient 2 (53 years)	Diff (%) Pat1 – Pat2	Patient 3
LVEDV (ml)	196	134	62 (−31.6 %)	316
LVESV (ml)	92.1	60.3	31.8 (−34.5 %)	249.6
LV mass (g)	141.3	165.6	−24.3 (−17.2 %)	108.5
LV-SV (ml)	103.9	73.7	30.2 (29.1 %)	66.4
LV-EF (%)	53	55	−2 (−3.8 %)	21
Heart rate (bpm)	57	55	2 (3.5 %)	77
Left atrium				
LA min volume (ml)	43.6	40.6	3 (6.9 %)	376
LA max volume (ml)	82.6	70.6	12 (14.5 %)	418
LA pre-contraction volume (ml)	64.4	61.9	2.5 (3.9 %)	--
Total conduit volume (ml)	39.0	30.0	9 (23.1 %)	42
Total LA EF (%)	47.2	42.5	4.7 (10.0 %)	10.0
Passive emptying volume (ml)	18.2	8.7	9.5 (52.2 %)	42
Active emptying volume (ml)	20.8	21.3	−0.5 (−2.4 %)	0
Passive emptying fraction (%)	47	29	18 (38.3 %)	100
Active emptying fraction (%)	53	71	−18 (−34.0 %)	0
Passive LA filling rate (ml/ms)	0.066	0.031	0.035 (53.0 %)	0.049
Active LA contraction rate (ml/ms)	0.067	0.109	−0.042 (−62.7 %)	--
LV stroke volume (ml)	104	73.7	30.3 (29.1 %)	66.4
“Passive LA flow” (ml)	65	43.7	21.3 (32.8 %)	24.4

### C. Intra- and inter-observer variability analysis

The variability analysis results are presented in mean difference ± SD format. Intra-observer agreement was high for both the minimal LA volume (−0.87 ± 1.65 ml or −1.2 ± 3.3 %) and the maximal LA volume (2.31 ± 3.07 ml or 2.1 ± 2.6 %). A high agreement was similarly found in inter-observer analysis for the minimal volumes (−0.01 ± 0.45 ml or −0.1 % ± 1.1 %) and maximal LA volumes (−0.12 ± 1.2 ml or −0.3 ± 1.1 %).

### D. CS-cineCMR image quality

Despite a high grade of image compression, the LA EBS of the CS-cineCMR images was not significantly different from EBS of standard non-compressed b-SSFP cine images at end diastole (long axis orientation: 0.039 ± 0.017 vs. 0.041 ± 0.03/pixel, *p* = 0.96; short axis orientation: 0.036 ± 0.02 vs. 0.04 ± 0.026/pixel, *p* = 0.863) and at end-systole (long axis orientation: 0.035 ± 0.012 vs. 0.055 ± 0.028/pixel, *p* = 0.31; short axis orientation: 0.036 ± 0.004 vs. 0.066 ± 0.034/pixel, *p* = 0.2).

## Discussion

In this study we present a novel multi-slice compressed-sensing cine MR technique combined with a non-model-based 3D reconstruction to measure the LA volumes during the whole cardiac cycle with high precision from a single breath-hold acquisition. This imaging strategy differs from conventional techniques of volume measurements, as it utilizes a new and highly accelerated compressed sensing MR strategy accelerated by compressed sensing and by its combination with a non-model based LA volume reconstruction method.

### Accuracy of CS-cineCMR for LA phantom volume measurement

This study validated the non-model-based 3D-reconstruction method developed by Bermano et al. [38] for phantoms mimicking typical LA shapes. When these various phantoms were covered by several non-parallel slices acquired during a single CS-cineCMR acquisition of approximatively 20-s duration, the 3D non-model-based reconstruction method yielded LA volumes which differed by only 4.7 ± 1.8 ml, i.e. 8.7 ± 3.5 %. Importantly, the standard deviation for these measurements was ±3.5 % (i.e. the 95 %-CI is ±6.6 %) for these LA volume measurements of different shapes (n = 15). In addition, when different scan plane orientations were applied (being aligned with the long-axis of the LA or not or acquiring short-axis slices through the LA’s perpendicular to the long-axis acquisition or not) the differences vs the references LA volumes were similar, which indicates that the 3D non-model-based reconstruction algorithm is precise even when slice orientations through the LAs vary. This has important practical implications, as it demonstrates that no specific planning rules need to be followed to allow for LA volume measurements of high accuracy.

The model-based, i.e. area-length-method, showed an underestimation in comparison to the true LA phantom volumes of 11.5 % which is in line with earlier studies reporting underestimation up to 20 % [15]. However, more importantly, the model-based biplane area-length approach was sensitive with respect to the alignment of long-axis acquisitions with the LA anatomy, which is likely to increase operator variability. The standard deviation of the difference between measured and true LA phantom volumes is an indicator whether the method’s accuracy depends on different LA shapes. Accordingly, the standard deviation of the new approach for LA volumes measurements was ±1.8 ml (i.e. ±3.5 %), whereas it was ±4.8 ml (i.e. ±8.5 %) for the model-based bi-plane area-length method. As this dependency of accuracy for the model-based area-length reconstruction was not found for the proposed non-model-based 3D reconstruction, this feature of the new approach facilitates planning of imaging planes and thus, is likely to reduce operator-dependence, and consequently, to increase reproducibility.

While non-invasive imaging techniques have been validated against LV phantoms, to our knowledge, no reports are available comparing non-invasive imaging methods against LA phantoms. In a study by Järvinen and coworkers, right atrial volumes measured by CMR were compared vs the true volume in atrial cadaveric casts and an underestimation of 7.2 ± 2.3 ml was found similar to the results reported here for the LA. However, complete volumetric coverage of the entire atrium necessitated a scan time of approximately 10 min [40]. Underestimation of volumes by non-invasive imaging techniques is not necessarily linked to 2D vs 3D acquisitions. In head-to-head comparisons using cardiac CT as a reference, several 3D trans-thoracic echocardiographic studies reported a significant underestimation of maximal LA volumes ranging from −8 to −48 % [41-44].

### Accuracy of CS-cineCMR for LA volume measurements in patients

Similar to the phantom measurements, we found an excellent agreement between the novel CS-cineCMR technique and the standard of reference with no significant difference in LA volumes with a mean difference of −2.7 ± 6.5 ml.

For the LA volume measurements in patients, the reference volumes were acquired with the ECG-triggered high-resolution self-navigated 3D-HR-CMR sequence [26]. This 3D self-navigation pulse sequence was validated in the LA phantoms and no difference was found in LA volumes compared to the true volumes determined by the water displacement technique (mean difference: −1.4 ± 1.4 ml; −2.4 ± 2.4 %, *p* = 0.08). Due to this validation, we accepted the high-resolution whole heart 3D datasets as the standard of reference for the LA volumes in the patients. This comparison demonstrated a high agreement of the novel CS-cineCMR technique with the 3D-high resolution data, when compared at the identical time point within the cardiac cycle. In contrast to the 3D-HR-CMR sequence, the CS-cineCMR technique yields complete time-volume curves of the LA which allows to derive various functional indices as shown in Fig. 5 and Table 5. Examples are shown in Fig. 6, where the top and the mid panel compare the LA time-volume curves of a 23 and 53 year-old patient. Although both patients were male with similar heart rates (57 vs 55 bpm) and LV ejection fractions (53 vs 55 %), their LA function was different with a smaller total reservoir volume (30 vs 39 ml) and a higher LA active emptying fraction (71 vs 53 %) in the older patient despite similar LA ejection fractions (43 vs 47 %). A population study recently highlighted the importance of the LA function, as it provided an incremental value over traditional cardiovascular risk factors to predict heart failure development [45]. In that study, however, LA volumes were measured on conventional long axis two and four-chamber cine views using the bi-plane area-length method. As the novel CS-cineCMR technique is not based on a model for LA volume measurements and the preliminary results demonstrate high accuracy in phantoms and in a small number of patients, there is potential for this novel approach to provide a more accurate estimate of the true LA volume changes over the cardiac cycle. This may in turn allow reducing the number of patients to be enrolled in future clinical studies.

### Intra and Inter observer variability

**Fig. 7:**
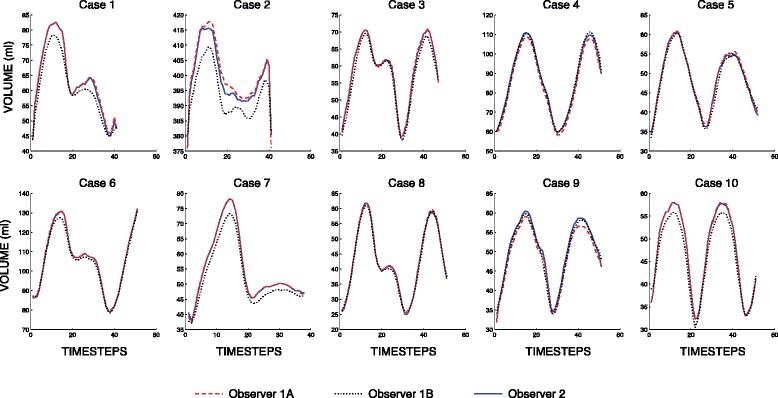
Graphical presentation of the time-volume curves derived from the evaluated compressed sensing cine datasets. In each graph, the three curves corresponding to the three segmentations are superimposed to illustrate the high reproducibility of the method

It was shown that the semi automatic segmentation does not induce any substantial variability in the corresponding estimation of LA volume. The intra- and inter-observer agreement was high with low values of mean differences and standard deviation of differences. Fig. 7 presents the time - volume curves for analyzed datasets as produced by the presented technique.

### Limitations

The segmentation of the datasets in this study was performed manually which can introduce observer variability. This potential variability was addressed by reassuring that the segmentations were performed by an experienced user. In the current study the accuracy of the CS-cineCMR technique was validated against the 3D-HR-CMR reference at a mid-diastolic phase only. However, as the LA contours on the CS-cineCMR images were readily visualized throughout all cardiac phases, it is assumed that the accuracy confirmed at the mid diastolic phase can be extrapolated to the whole time-volume curve.

The results presented in this study refer to acquisitions on a 1.5 T scanner and cannot be extrapolated directly to higher field strengths.

## Conclusion

We describe a novel strategy for the measurement of LA volumes based on a highly accelerated compressed sensing cine CMR acquisition performed during one single breath hold which is combined with a non-model-based 3D reconstruction method. From five 2D cine-slices, the LA geometry is reconstructed for each phase of the cardiac cycle. The calculated volumes showed an excellent agreement with the reference volumes in phantoms and patients. Time volume-curves were easily derived to assess the LA function and the reproducibility was high as analyzed on a small sample of patients. Further validation on a larger population is needed.
